# Supplementary material for: High Resistance to Antibiotics Recommended in Standard Treatment Guidelines in Ghana: A Cross-Sectional Study of Antimicrobial Resistance Patterns in Patients with Urinary Tract Infections between 2017–2021
Source: Int J Environ Res Public Health. 2022 Dec 9;19(24):16556. doi: 10.3390/ijerph192416556 (PMC9779193; doi:10.3390/ijerph192416556)
Supplement: Supplementary file 1 [file ijerph-19-16556-s001.zip › ijerph-1956434-supplementary.pdf]

Supplementary Materials:

Supplementary Table S1: Total number of all bacteria isolates at MDS lancet laboratories Ghana from 2017 and 2021

BACTERIA		SEX		TOTAL
GNB	MALES N(%)	FEMALE N(%)	UNKNOWN N(%)	N (%)
Aeromonas hydrophila	1 (0.02)	0 (0.00)	0 (0.00)	1 (0.00)
Acinetobacter baumannii	20 (0.35)	25 (0.17)	0 (0.00)	45 (0.22)
Acinetobacter lwoffii	1 (0.02)	2 (0.01)	0 (0.00)	3 (0.01)
Citrobacter fruendii	99 (1.76)	91 (0.61)	1 (4.17)	191 (0.93)
Citrobacter brakii	4 (0.07)	7 (0.05)	0 (0.00)	11 (0.05)
Citrobacter diversus	8 (0.14)	26 (0.18)	0 (0.00)	34 (0.17)
Citrobacter farmeri	1 (0.02)	2 (0.01)	0 (0.00)	3 (0.01)
Citroacter spp	5 (0.09)	9 (0.06)	0 (0.00)	14 (0.07)
citrobacter youngae	12 (0.21)	18 (0.12)	0 (0.00)	30 (0.15)
Escherichia coli	3,552 (62.97)	11,129 (74.94)	14 (58.33)	14,695 (71.63)
Escherichia vulneris	1 (0.02)	0 (0.00)	0 (0.00)	1 (0.00)
Enterobacter aerogenes	52 (0.92)	141 (0.95)	0 (0.00)	193 (0.94)
Enterobacter amnigenus 1	2 (0.04)	6 (0.04)	0 (0.00)	8 (0.04)
Enterobacter amnigenus 2	0 (0.00)	4 (0.03)	0 (0.00)	4 (0.02)
Enterobacter asburiae	0 (0.00)	2 (0.01)	0 (0.00)	2 (0.01)
Enterobacter cloacae	286 (5.07)	261 (1.76)	0 (0.00)	547 (2.67)
Enterobacter gergoviae	4 (0.07)	8 (0.05)	0 (0.00)	12 (0.06)
Enterobacter spp	1 (0.02)	0 (0.00)	0 (0.00)	1 (0.00)
hafnia alvei	0 (0.00)	6 (0.04)	0 (0.00)	6 (0.03)
Klebsiella oxytoca	21 (0.37)	46 (0.31)	0 (0.00)	67 (0.33)
klebsiella ozaenae	5 (0.09)	7	0 (0.00)	12 (0.06)
klebsiella pneumoniae	1,090 (19.32)	1,933 (13.02)	5 (20.83)	3,028 (14.76)
Klebsiella rhinoscleromatis	1 (0.02)	0 (0.00)	0 (0.00)	1 (0.00)
Klebsiella spp	1 (0.02)	3 (0.02)	0 (0.00)	4 (0.02)
Kluyvera ascorbata	0 (0.00)	1 (0.01)	0 (0.00)	1 ((0.00)
kluyvera spp	15 (0.27%)	21 (0.14)	0 (0.00)	36 (0.18)
Morganella morganii	21 (0.37)	36 (0.24)	0 (0.00)	57 (0.28)
Pantoea agglomerans	1 (0.02)	2 (0.01)	0 (0.00)	3 (0.01)
Pantoea spp	14 (0.25)	26 (0.18)	0 (0.00)	40 (0.19)
Proteus mirailis	228 (4.04)	862 (5.8)	3 (12.50)	1,093 (5.33)

Proteus penneri	5 (0.09)	3 (0.02)	0 (0.00)	8 (0.4)
Proteus vulgaris	7 (0.12)	10 (0.07)	0 (0.00)	17 (0.08)
Proteus spp	0 (0.00)	2 (0.01)	0 (0.00)	2 (0.01)
Providencia alcalifaciens	0 (0.00)	1 (0.01)	0 (0.00)	1 (0.00)
Providencia rettgeri	22 (0.39)	0 (0.00)	0 (0.00)	22 (0.11)
Providencia rustigianii	1 (0.02)	2 (0.01)	0 (0.00)	3 (0.01)
Providencia spp	1 (0.02)	0 (0.00)	0 (0.00)	1 (0.00)
Providencia stuartii	8 (0.140	5 (0.03)	0 (0.00)	13 (0.06)
Pseudomonas aeruginosa	53 (0.94)	36 (0.24)	1 (4.17)	90 (0.44)
Pseudomonas fluorescens	4 (0.07)	5 (0.03)	0 (0.00)	9 (0.04)
Pseudomoas luteola	0 (0.00)	1 (0.01)	0 (0.00)	1 (0.00)
Raoultella ornithinolytica	17 (0.30)	37 (0.250	0 (0.00)	54 (0.26)
Serratia ficaria	9 (0.16)	12 (0.08)	0 (0.00)	21 (0.10)
Serratia fonticola	8 (0.14)	8 (0.05)	0 (0.00)	16 (0.08)
Serratia liquefaciens	13 (0.23)	13 (0.09)	0 (0.00)	26 (0.13)
Serratia marcescens	29 (0.51)	21 (0.14)	0 (0.00)	50 (0.24)
Serratia odorifera	8 (.014)	12 (0.08)	0 (0.00)	20 (0.10)
Serratia plymuthica	5 (0.09)	7 (0.05)	0 (0.00)	12 (0.06)
Serratia rubidaea	3 (0.05)	0 (0.00)	0 (0.00)	3 (0.01)
Stenotrophomonas maltophilia	2 (0.04)	1 (0.01)	0 (0.00)	3 (0.01)
**TOTAL N(%)**	5,641 (100)	14,850 (100)	24 (100)	20,515 (100)
**GPC**				
Enterococcus faecalis	40 (90.91)	227 (90.44)	0 (0.00)	267 (90.51)
Enterococcus faecium	2 (4.55)	0 (0.00)	0 (0.00)	2 (0.68)
Enterococcus spp	0 (0.00)	1 (0.40)	0 (0.00)	1 (0.34)
Staphylococcus aureus	0 (0.00)	5 (1.99)	0 (0.00)	5 (1.69)
Staphylococcus saprophyticus	2 (4.55)	9 (3.59)	0 (0.00)	11 (3.73)
Streptococcus group B	0 (0.00)	9 (3.59)	0 (0.00)	9 (3.05)
**TOTAL N(%)**	44 (100)	251 (100)	0 (0.00)	295 (100)

[1]GPC: Gram positive cocci

[2]GNB: gram negative bacilli

Supplementary Table S2: The susceptibility patterns of tested antibiotics against ESBL positive bacteria at the MDS-lancet laboratories Ghana from 2017 to 2021

Antibiotic	ESBL+			Total
	S	R	N/A	
Amikacin	9,273 (94.61)	522 (5.33)	6 (0.06)	9,801
Ciprofloxacin	1,340 (13.67)	8,425 (85.96)	36 (0.37)	9,801
Cotrimoxazole	862 (8.80)	8,791 (89.69)	148 (1.51)	9,801
Ertapenem	9,591 (97.86)	203 (2.07)	7 (0.07)	9,801
Fosfomycin	8,937 (91.18)	529 (5.40)	335 (3.42)	9,801
Gentamycin	4,108 (41.91)	5,693 (58.09)	0 (0.00)	9,801
Imipenem	9,565 (97.59)	113 (1.15)	123 (1.25)	9,801
Meropenem	9,601 (97.96)	124 (1.27)	76 (0.78)	9,801
Nitrofurantoin	5,648 (57.63)	4,077 (41.60)	76 (0.78)	9,801
Tegicyclin	7,803 (79.61)	462 (4.71)	1,536 (15.67)	9,801

N/A: not applicable means that antibiotic was not reported for the isolate or was not available for the isolated or is not tested at all for the isolate and as such not reported.
